# Social medicine’s long history

**DOI:** 10.1590/S0104-59702024000100066

**Published:** 2024-12-16

**Authors:** Jonathan D. Ablard

**Affiliations:** i Department of History/Ithaca College.Ithaca – NY – USA jablard@ithaca.edu

Geographer Eric D. Carter has produced a thorough and compelling analysis of the history of social medicine in Latin America. His work tells a synthetic region-wide history that manages to pay attention to national case studies and trends that cross borders.

Carter (2023, p.3) defines social medicine as an “academic field that principally focuses on structural determinants of health and illness … from diverse disciplinary perspectives [and] takes a critical stance toward mainstream ideas and practices in medicine and health policy.” Advocates of social medicine, even before the term itself existed, have tended to be politically engaged in the struggle to achieve health equity.

On the historiographic level, Carter’s is a synthesis of “national-scale” studies of social medicine. The author challenges the notion that Latin American social medicine derived exclusively from European models; similarly, Carter demonstrates that ideas and projects which we now think of as social medicine were not the sole purview of the left. This book is populated by doctors and health professionals from across the political spectrum who created influential networks and institutions of national and international relevance. Violent regime change, the biomedical and bureaucratic imperatives of post-Second World War international agencies, as well as resistance to comprehensive health systems that came from predictable and surprising sectors, all presented challenges to improving health outcomes.

In chapter one, Carter argues for the diverse origins of Latin American social medicine. Central to its development starting in the late nineteenth century was the “social question,” which we can characterize as a body of concepts, proposals, and policies designed to address the myriad problems wrought of “capitalist modernization.” Doctors came to these debates from anarchism, socialism, traditional conservatism, and the Catholic teachings of *Rerum novarum*.

Chapter two examines the relationship between, and influence of, the League of Nations Health Organization, the International Labor Office, the Rockefeller Foundation, and the Pan American Health Organization. Echoing a growing body of scholarship on science and medicine in Latin American history, Carter sees the exchange of ideas as a multi-directional process (Birn, Necochea López, 2020). “By and large, Latin American experts favored ‘internationalism,’ in the sense of exchanging ideas across borders, but they were skeptical of ‘universalism,’ the notion that good policies would be effective anywhere” (Carter, 2023, p.66). The model of the US New Deal was consequently received with great interest, while by the 1930s eugenic models from the global north were increasingly viewed with disinterest.

Chapters three and four examine the relative success of health policy reforms in Chile and Argentina. For Chile, Carter (2023, p.70) makes a good case that “social medicine was a relatively open domain that encompassed varied analytics of the political, economic, and social causes of health crisis.” And just as conservative doctors made important contributions to both ideas and practices, unions were not necessarily supportive of wider health reforms, which they sometimes feared would encroach upon their autonomy. Ideological pluralism in the field of social medicine, Carter argues, “helps explain why, when the legislation that would eventually create the Servicio Nacional de Salud (SNS) was introduced to the national congress in 1950,” deputies from across the Chilean political spectrum supported it (p.91).

Chapter four explains “the incomplete development of the Argentine welfare state and the lasting fragmentation of the health system” (Carter, 2023, p.95). As in Chile, Argentina’s social medicine story involved contributions by professionals and activists with diverse political orientations. Two major factors, however, impeded national level reforms. One was divisive politics, especially the banning of centrist and leftist political parties after the 1930 coup. The other challenge was opposition to a national health system from a host of very different associations including organized labor, women’s organizations, clientelist political parties, and doctors’ groups, all seeking to defend their collective prerogatives. Even the Perón government of 1946-1955 only enjoyed “mixed success” against this array of opponents to a unified system.

Regionally, one of the greatest challenges to social medicine’s development in the post-1945 period was the growing influence of modernization theory and the international health organizations that subscribed to its tenets. Chapter five outlines the complexities of navigating the political and professional pressures that accompanied the Cold War. The period also witnessed outright domestic persecution of social medicine advocates in Chile. But in Brazil, which was also under a military regime, the influence of social medicine grew rather than diminished as its approach aligned with certain development goals of the government.

Social medicine enjoyed a revival in the 1970s and 1980s which culminated in the creation of the Latin American Social Medicine Association (Alames). The creation of new networks and introduction of novel theoretical approaches occurred during a period of political repression in many of the countries that had been leaders in social medicine. Argentine Juan César Garcia’s leadership in the Pan American Health Organization’s medical education unit was critical to organize “a network of scholars and health workers interested in developing cutting-edge social science research to be applied to the health field” (Carter, 2023, p.152). The 1973 coup in Chile sent hundreds of doctors into exile; Carter charts the movement of these men and women and the influence they had in their new professional lives. While Brazil was also subject to military rule, the regime took a decentralized approach and “*saúde coletiva* could thrive in municipalities where progressive ideas were tolerated” (p.169). Finally, health professionals began to incorporate a new set of intellectual currents including liberation pedagogy, postcolonialism, feminism, and the writings of dependency theorists. All in all, members and followers of Alames began to envision healthcare outside of the rigid confines of biomedical and technocratic frameworks.

The return of democracy in the Southern Cone region brought with it both openings for social medicine as well as new challenges from the debt crisis and ensuing neoliberal reforms. While Brazil instituted its still very successful Unified Health System [Sistema Único de Saúde], other countries struggled to stem the tide of social spending cuts. New opportunities emerged during the Pink Tide of the late 1990s, and health professionals aligned with Alames moved into high-level government positions.

Social medicine has had an outsized impact on health systems and outcomes in Latin America, a region characterized by high levels of income inequality and periods of political repression and economic instability. Social medicine’s successes have partly resulted from a tradition of networking across both disciplines and national borders, a willingness to engage with diverse social and political actors, and “the organized interests of the health sector” (Carter, 2023, p.217).

Carter suggests new future research paths to readers, calling for greater attention to the role of networks and the development of the social science of health in the region. I would also suggest that more work is needed to understand the role race has played in medical ideas from the second half of the twentieth century to the present. Race disappears from *In pursuit* by the 1930s. Were doctors simply ignoring it? Or were they thinking of class and race as largely interchangeable (Green, McKiernan-Gonzalez, Summers, 2014)? Finally, Carter’s is a heavily male story, but by the 1980s and 1990s a number of women had reached important positions in national health care systems. Their stories will add an important dimension to understanding the ongoing evolution of social medicine in both theory and practice.
